# Correction: Eshaq et al. Non-Receptor Tyrosine Kinases: Their Structure and Mechanistic Role in Tumor Progression and Resistance. *Cancers* 2024, *16*, 2754

**DOI:** 10.3390/cancers18203261

**Published:** 2026-10-10

**Authors:** Abdulaziz M. Eshaq, Thomas W. Flanagan, Sofie-Yasmin Hassan, Sara A. Al Asheikh, Waleed A. Al-Amoudi, Simeon Santourlidis, Sarah-Lilly Hassan, Maryam O. Alamodi, Marcelo L. Bendhack, Mohammed O. Alamodi, Youssef Haikel, Mossad Megahed, Mohamed Hassan

**Affiliations:** 1 Department of Epidemiology and Biostatistics, Milken Institute School of Public Health, George Washington University, Washington, DC 20052, USA; 2 College of Medicine, Alfaisal University, Riyadh 11533, Saudi Arabia; 3 Department of Pharmacology and Experimental Therapeutics, LSU Health Sciences Center, New Orleans, LA 70112, USA; 4 Department of Pharmacy, Faculty of Science, Heinrich-Heine University Duesseldorf, 40225 Duesseldorf, Germany; 5 Institute of Cell Therapeutics and Diagnostics, University Medical Center of Duesseldorf, 40225 Duesseldorf, Germany; 6 Department of Chemistry, Faculty of Science, Heinrich-Heine University Duesseldorf, 40225 Duesseldorf, Germany; 7 Department of Urology, Red Cross University Hospital, Positivo University, Rua Mauá 1111, Curitiba 80030-200, Brazil; 8 Institut National de la Santé et de la Recherche Médicale, University of Strasbourg, 67000 Strasbourg, France; 9 Department of Operative Dentistry and Endodontics, Dental Faculty, 67000 Strasbourg, France; 10 Pôle de Médecine et Chirurgie Bucco-Dentaire, Hôpital Civil, Hôpitaux Universitaire de Strasbourg, 67000 Strasbourg, France; 11 Clinic of Dermatology, University Hospital of Aachen, 52074 Aachen, Germany; 12 Research Laboratory of Surgery-Oncology, Department of Surgery, Tulane University School of Medicine, New Orleans, LA 70112, USA

## Text Correction

There was an error in the original publication [1]. The following sentence “The oncogenic activation of Abl1 in BCR-ABL1 chimeric proteins is mediated by an N-terminal coiled-coil (CC) oligomerization-dependent mechanism [226].” has been revised and replaced by the following sentence, “The oncogenic activation of Abl1 in BCR-ABL1 chimeric proteins is indeed mediated by an N-terminal coiled-coil (CC) domain, which acts as an oligomerization-dependent mechanism to trigger constitutive kinase activity [231,232]”.

The following sentence “The oncogenic activity of Abl1 in the context of the ABL1-BCR fusion protein is mediated via the BCR N-terminal coiled-coil (CC) oligomerization domain [208,239,240]” has been replaced by the following, “The oncogenic activity of the BCR-ABL1 fusion protein is primarily mediated through the N-terminal coiled-coil (CC) oligomerization domain of the BCR portion, which brings multiple ABL1 kinase domains into close proximity, leading to constitutive tyrosine kinase activation [241,242]” together with the supported references.

## Extra Citation

The following reference should be removed:

Luttman, J.H.; Colemon, A.; Mayro, B.; Pendergast, A.M. Role of the ABL tyrosine kinases in the epithelial-mesenchymal transition and the metastatic cascade. *Cell Commun. Signal.* **2021**, *19*, 59.

## Missing Citation

The newly added references appear below:

231. Beissert, T.; Puccetti, E.; Bianchini, A.; Güller, S.; Boehrer, S.; Hoelzer, D.; Ottmann, O.G.; Nervi, C.; Ruthardt, M. Targeting of the N-terminal coiled coil oligomerization interface of BCR interferes with the transformation potential of BCR-ABL and increases sensitivity to STI571. *Blood* **2003**, *102*, 2985–2993.
232. Massimino, M.; Stella, S.; Romano, C.; Buffa, P.; Tirrò, E.; Drago, M.; Manzella, L.; Tomarchio, C.; Vitale, S.R.; Raimondo, F.D.; et al. The Coiled Coil and C2 Domains Modulate BCR Localization and BCR-ABL1 Compartmentalization, Transforming Activity and TKI Responsiveness. *Int. J. Mol. Sci.* **2025**, *26*, 6591.
241. Shammas, T.; Peiris, M.N.; Meyer, A.N.; Donoghue, D.J. BCR-ABL: The molecular mastermind behind chronic myeloid leukemia. *Cytokine Growth Factor Rev.* **2025**, *83*, 45–58.
242. Peiris, M.N.; Li, F.; Donoghue, D.J. BCR: A promiscuous fusion partner in hematopoietic disorders. *Oncotarget* **2019**, *10*, 2738–2754.

With this correction, the order of some references has been adjusted accordingly. The authors apologize for any inconvenience caused and state that the scientific conclusions are unaffected. This correction was approved by the Academic Editor. The original publication has also been updated.
